# Overexpression of DHX32 contributes to the growth and metastasis of colorectal cancer

**DOI:** 10.1038/srep09247

**Published:** 2015-03-18

**Authors:** Huayue Lin, Wenjuan Liu, Zanxi Fang, Xianming Liang, Juan Li, Yongying Bai, Lingqing Lin, Hanyu You, Yihua Pei, Fen Wang, Zhong-Ying Zhang

**Affiliations:** 1 Center for Clinical Laboratory, Xiamen University Affiliated Zhongshan Hospital, Xiamen, China; 2 State Key Laboratory of Molecular Vaccinology and Molecular Diagnostics, School of Public Health, Xiamen University, Xiamen, China; 3 Central Laboratory, Xiamen University Affiliated Zhongshan Hospital, Xiamen, China; 4 Center for Cancer and Stem Cell Biology, Institute of Biosciences and Technology, Texas A&M Health Science Center, Houston, TX

## Abstract

Our previous work demonstrates that DHX32 is upregulated in colorectal cancer (CRC) compared to its adjacent normal tissues. However, how overexpressed DHX32 contributes to CRC remains largely unknown. In this study, we reported that DHX32 was overexpressed in human colon cancer cells. Overexpressed DHX32 promoted SW480 cancer cells proliferation, migration, and invasion, as well as decreased the susceptibility to chemotherapy agent 5-Fluorouracil. Furthermore, PCR array analyses revealed that depleting DHX32 in SW480 colon cancer cells suppressed expression of *WISP1*, *MMP7* and *VEGFA* in the Wnt pathway, and anti-apoptotic gene *BCL2* and *CA9*, however, elevated expression of pro-apoptotic gene *ACSL5.* The findings suggested that overexpressed DHX32 played an important role in CRC progression and metastasis and that DHX32 has the potential to serve as a biomarker and a novel therapeutic target for CRC.

Colorectal cancer (CRC) is one of the leading causes of cancer-related deaths with high morbidity and mortality^1^. The high mortality nature of colon cancer is due to its sporadic origin and diagnosed at advanced stages. 5-Fluorouracil (5-FU) has been used for more than 40 years in treating various cancers and remains as the standard first-line chemotherapeutic drug for CRCs, although as high as 50% of metastatic CRCs resist to 5-FU-based chemotherapies^2^,^3^,^4^. Therefore, understanding the molecular basis of genetic and epigenetic changes that contribute to CRC progression and the targets of 5-FU in CRC are important for developing new therapeutic strategies and for overcoming drug resistance.

RNA helicases are members of the DExD/H-box family, which are characterized by the presence of a helicase domain and are involved in RNA metabolism^5^,^6^. In addition to their roles in RNA metabolism, multiple members of the DExD/H-proteins are also implicated in transcription regulations, including RNA helicase A (DHX9)^7^ and p68 (DHX5)^8^,^9^. Several of RNA helicases are dysregulated in cancer cells, although the exact contributions of RNA helicases to cancer initiation and progression have not been fully characterized^10^,^11^. DHX32 is originally identified as a novel RNA helicase with the unique helicase domain^12^. DHX32 has a widespread tissue distribution. Human and murine DHX32 exhibits a high similarity in the amino acid sequences, suggesting that it is a functionally important and well-conserved gene^12^,^13^. However, the functions of DHX32 are largely unknown. We reported previously that the expression of *DHX32* is up-regulated in CRCs compared to its adjacent normal tissues and that the level of *DHX32* expression is associated with cancer location, lymph gland metastasis, cancer nodal status, differentiation grade, and Dukes' stage^14^. In this study, we investigated the function of DHX32 in CRC cells and systematically examined gene expression profile changes in major signal transduction pathways affected by depleting DHX32. The data showed that DHX32 promoted proliferation, migration, and invasion of CRC cells, as well as reduced sensitivity for 5-FU treatment. Furthermore, our data also showed that DHX32 upregulated the Wnt pathway and downregulated pro-apoptotic gene expression. The results suggested the potential of DHX32 as a biomarker for CRC diagnosis and as a novel target for CRC treatment.

## Results

### Upregulated expression of DHX32 in human CRC cells

**Figure 1:**
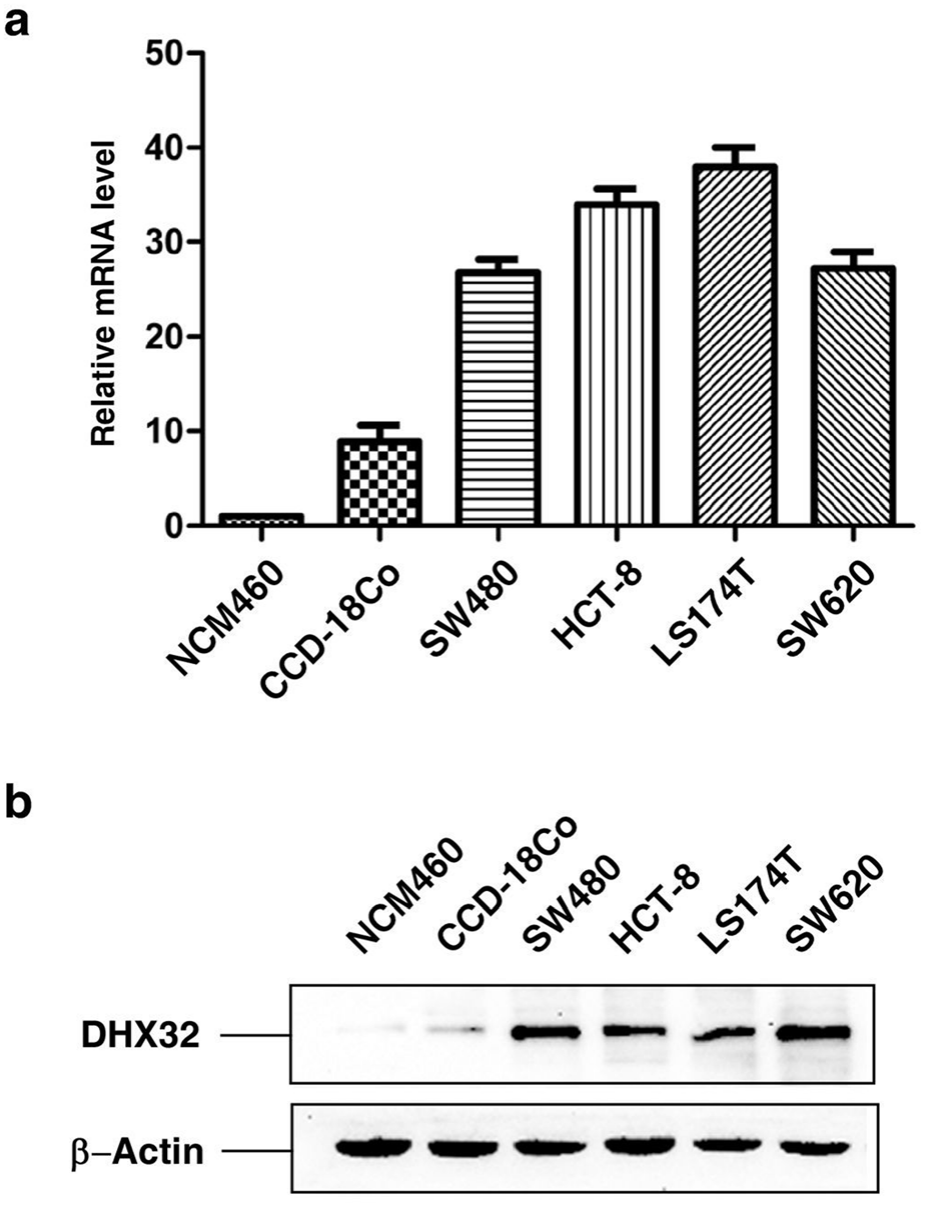
DHX32 is overexpressed in CRCs compared to normal human colonic epithelial cell. Expression of DHX32 was determined with real-time RT-PCR (a) or western blot (b) analyses (the blots were cropped, and the full-length blots were included in the supplementary information). Note that DHX32 expression was higher in SW480, HCT-8, LS174T, and SW620 than in NCM460 and CCD-18Co cells. β-actin levels were used as a loading control.

RNA helicase DHX32 is highly expressed in human CRC compared to its adjacent normal tissues^14^. To further study whether DHX32 was also overexpressed in human CRC cells, quantitative real-time RT-PCR (Fig. 1a) and Western blot (Fig. 1b) analyses were carried out to assess the expression of DHX32 in human CRC cell lines SW480, HCT-8, LS174T and SW620, as well as normal human colonic epithelial cell lines NCM460 and CCD-18Co at the mRNA level and protein level, respectively. Consistent with our previous report in human colorectal cancer samples, expression of DHX32 at both the mRNA and protein levels was higher in human CRC cells than in normal human colonic epithelial cells.

### Overexpression of DHX32 promotes proliferation of CRC cells

To unravel the role of DHX32 in CRC tumorigenicity, DHX32 was depleted or overexpressed by stable expression of DHX32 specific shRNA or full-length DHX32 cDNA, respectively. Both real-time RT-PCR and Western analyses demonstrated that expression of DHX32 was reduced by shRNA or increased by cDNA transfection, respectively (Fig. S1).

**Figure 2:**
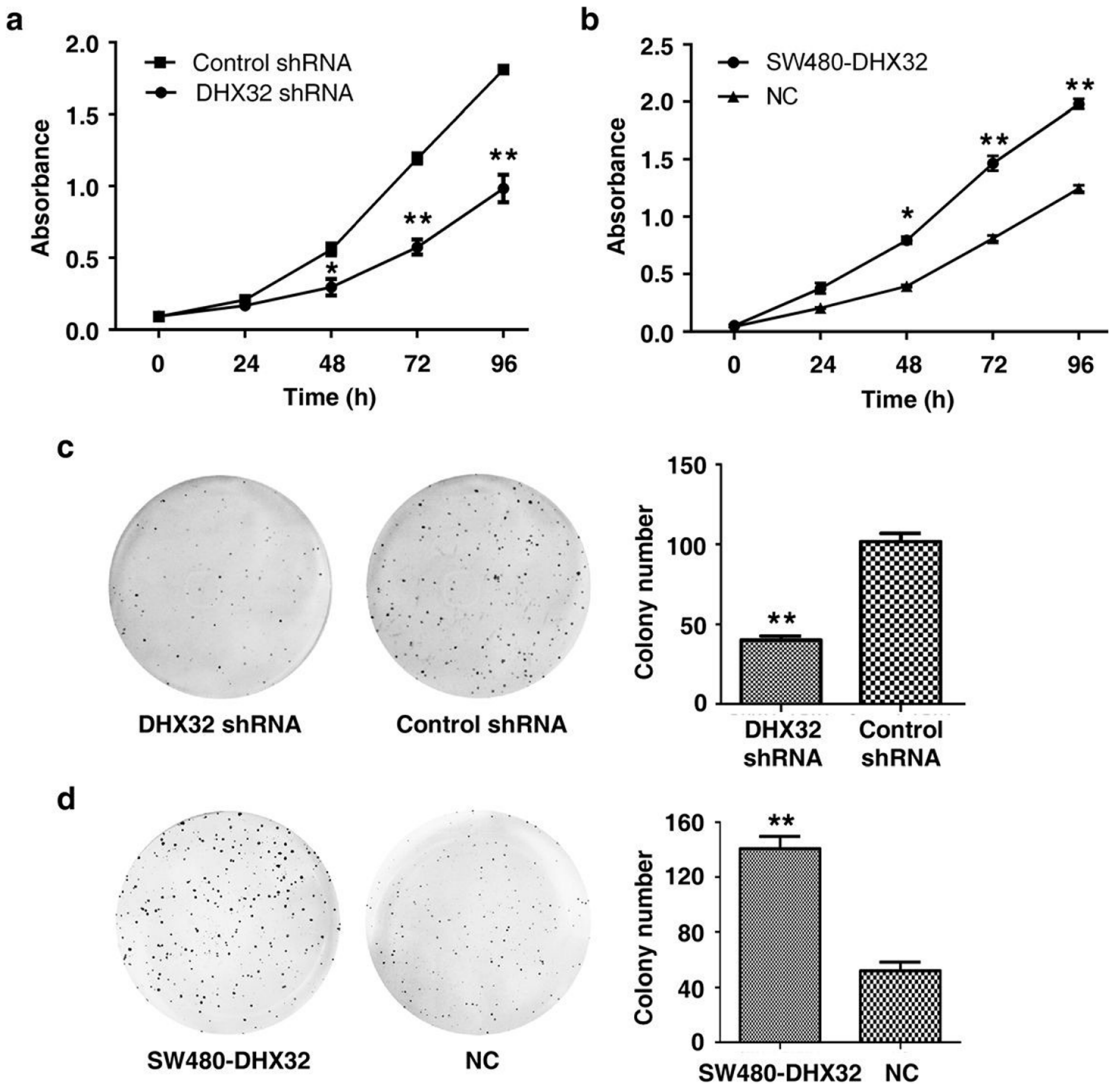
DHX32 increases proliferation and colony formation of CRC cells. (a&b), DHX32 expression level is associated with cell proliferation of SW480 cells. DHX32 depleted (a) or overexpressed (b) and the control SW480 cells were cultured for 24, 48, 72, and 96 hours. The cell density was then determined with the CCK-8 Assay Kit as described in Methods. (c&d), DHX32 expression level is associated with colony formation activity of SW480 cells. Cell colonies derived from DHX32 depleted (c) or overexpressed (d) SW480 cells were counted. Data are mean ± SD from triplicated samples. NC, vector only transfectant as a negative control; *P<0.05, **P<0.01.

To determine whether the expression level of DHX32 affected CRC cell proliferation, the Cell Counting Kit-8 (CCK-8) was employed to assess CRC cell growth with or without depletion or overexpression of DHX32. The results showed that the DHX32-depleted cells exhibited a decreased growth rate compared to that of control cells, whereas overexpression of DHX32 increased cell proliferation (Fig. 2a&2b). Consistently, colony formation assays demonstrated that the number of colonies was increased in DHX32-overexpressed and reduced in DHX32-depleted SW480 cell groups (Fig. 2c&2d). The results suggest that DHX32 contributes to proliferation and colony formation of CRC cells.

### Overexpressed DHX32 reduces 5-FU induced apoptosis in CRC cells

**Figure 3:**
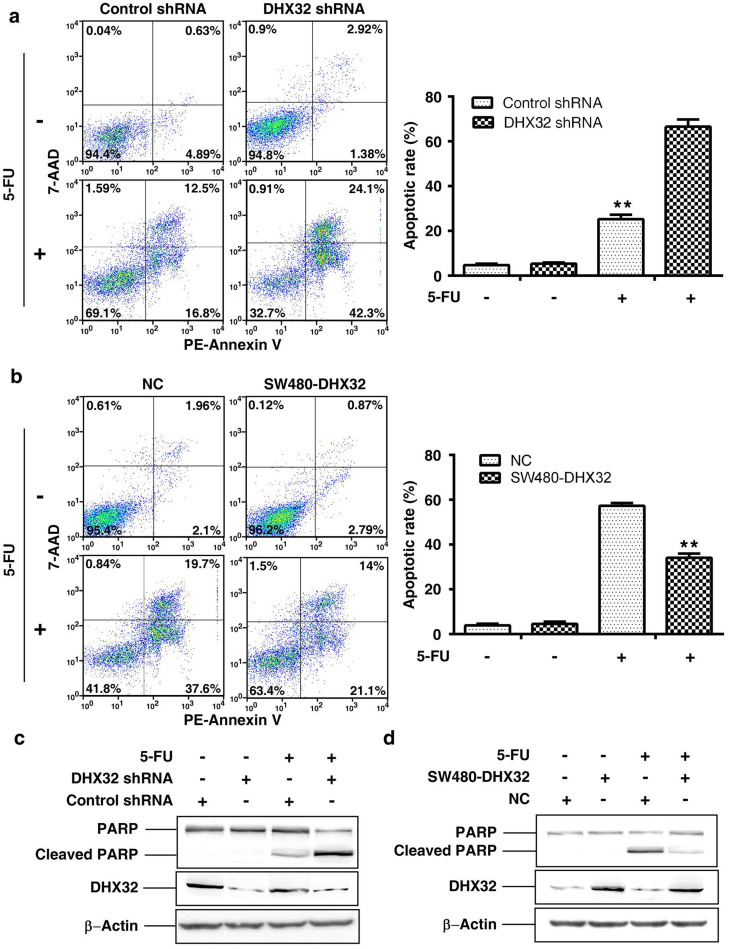
DHX32 reduces 5-FU induced apoptosis in CRC cells. (a&b), Annexin V labeling based flow cytometry analyses of apoptotic cells. DHX32 depleted and control SW480 (a) were subjected to 20 μM 5-FU treatment. DHX32 overexpressed and negative control SW480 (b) were subjected to 40 μM 5-FU treatment. Apoptotic cells were detected with a flow cytometry at 48 hours after the treatment. Early and late stages of apoptosis were identified as Annexin V^+^/7-AAD^−^ and Annexin V^+^/7-AAD^+^, respectively. (c&d), Western blot analyses showed that the PARP cleavage was increased in DHX32 depleted (c) or decreased in DHX32 overexpressed (d) SW480 cells at 36 hours after being treated with 5-FU (the blots were cropped, and the full-length blots were included in the supplementary information). NC, vector only as a negative control; **P<0.01.

To determine whether depletion or overexpression of DHX32 affects cell viability, the cells were labeled with anti-Annexin V and 7-AAD and then subjected to fluorescent assisted cell sorting (FACS) analyses. The results clearly demonstrated that neither overexpression nor depletion of DHX32 changed apoptotic cell population in SW480 cells. It has been shown that DHX32 desensitize T-cells from responding to apoptotic stimuli^15^. 5-FU is a commonly used apoptosis-inducing drug in CRC treatment. To investigate whether DHX32 protected CRC undergoing apoptosis induced by 5-FU, SW480 cells were treated with 5-FU in the culture medium. As expected, 5-FU induced notable changes in cell morphology and density, especially in DHX32-depleted cells. In order to assess the cell numbers at different apoptotic stages, the cells were stained with anti-Annexin V and 7-AAD, the early and late apoptotic markers, respectively. As shown in Figure 3a, treating SW480 cells with 5-FU resulted in an increase in apoptosis. Depletion of DHX32 increased both Annexin V and 7-AAD labeled cell populations, whereas overexpression of DHX32 reduced both cell populations (Fig. 3b).

To further assess the involvement of DHX32 in 5-FU induced apoptosis, we analyzed apoptosis-related protein poly ADP-ribose polymerase (PARP) expression in 5-FU treated cells. Indeed, depletion of DHX32 expression increased the cleavage of PARP in 5-FU treated SW480 cells (Fig. 3c), and overexpression of DHX32 diminished the cleavage of PARP in 5-FU treated SW480 cells (Fig. 3d). Taken together, the results demonstrate that although the expression level of DHX32 per se does not affect cell apoptosis, it modulates the response to apoptotic reagents in CRC cells although the underlying molecular mechanism remains to be unveiled.

### DHX32 enhances migration and invasion of CRC cells

**Figure 4:**
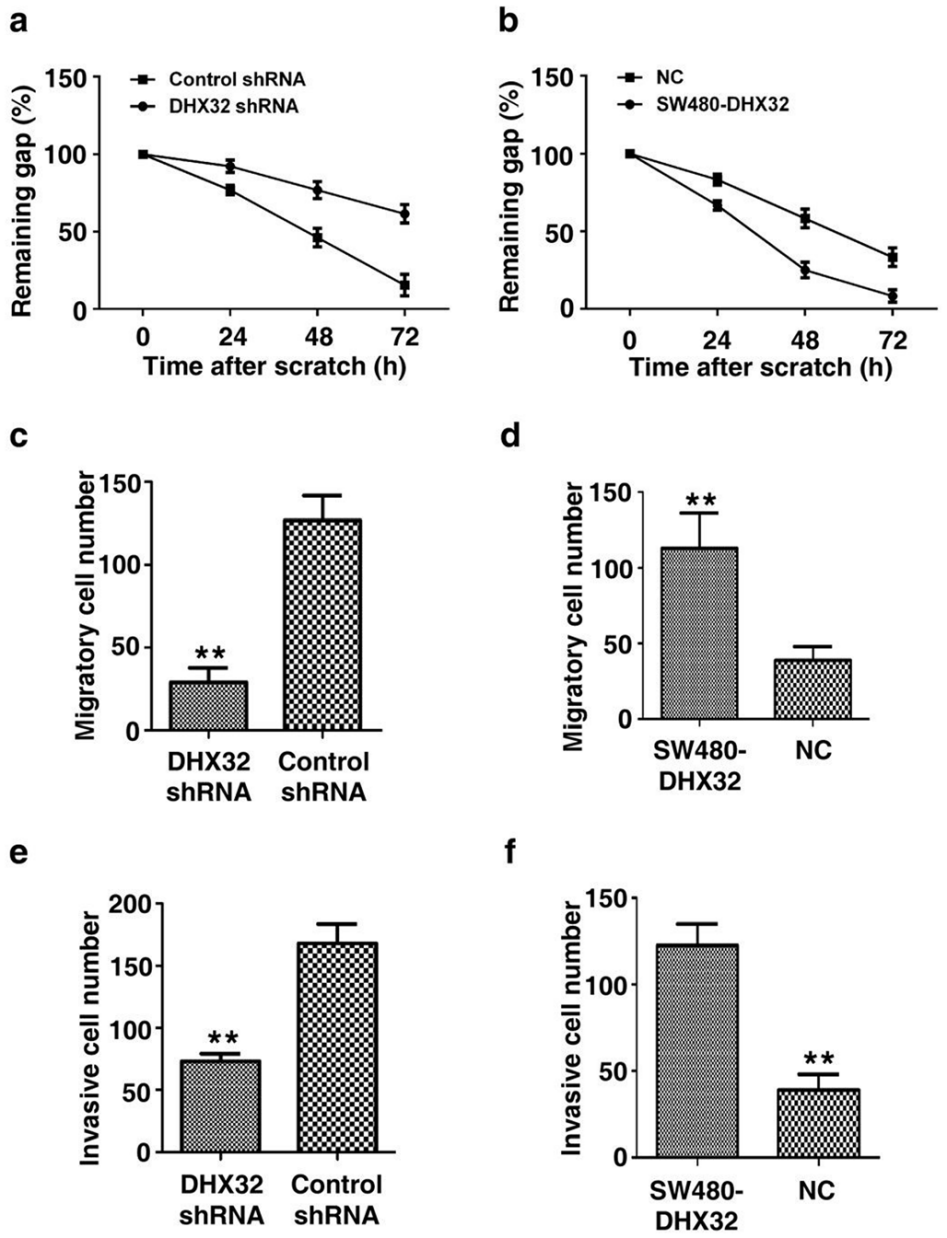
DHX32 promotes CRC cells migration and invasion. (a&b), Scratch wound assays for DHX32 depleted (a) or overexpressed (b) SW480 cells. The average sizes of the gaps were measured at the indicated times and expressed as mean ± SD of triplicated samples. (c&d), Transwell chamber assays for DHX32 depleted (c) or overexpressed (d) SW480 cells. The average numbers of migrated cells were counted after 24-hour incubations and expressed as mean ± SD. (e&f), Transwell Matrigel invasion assays for DHX32 depleted (e) or overexpressed (f) SW480 cells. The average numbers of migrated cells were quantitated after 24-hour incubations and expressed as mean ± SD. NC, vector only as a negative control; **P<0.01.

To determine whether the expression level of DHX32 affected cell motility, the scratch wound-healing assay was carried out. As shown in Fig. 4a and Fig. S2a, the scratch gap in the control cells was almost half-closed within 48 hours. However, the scratch gap in the DHX32-depleted cells was not closed even at day 3. In contrast, DHX32-overexpressed clones displayed more cells in the gap than in the control cells (Fig. 4b & Fig. S2b). Similarly, the Transwell assay also demonstrated that depletion of DHX32 compromised cell migration activity (Fig. 4c & Fig. S2c), whereas DHX32 overexpression resulted in enhancing transmembrane migration activity of the cells (Fig. 4d & Fig. S2d). Furthermore, the transwell Matrigel invasion assay also showed that depletion of DHX32 expression reduced invasion activities (Fig. 4e & Fig. S2e) and overexpression of DHX32 enhanced the invasion activity of SW480 cells (Fig. 4f & Fig. S2f). Together, the results demonstrate that DHX32 contributes to the migration and invasion activities of CRC cells.

### DHX32 contributes to the regulation of genes important for cell migration and apoptosis

**Figure 5:**
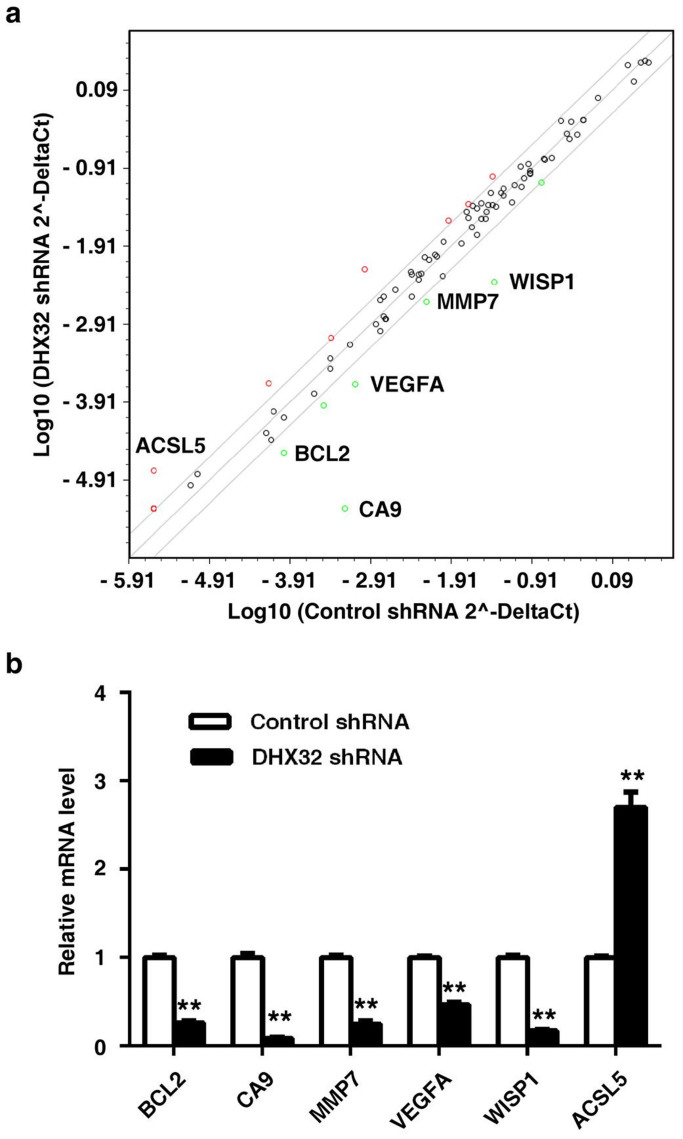
Depleting DHX32 induces gene expression changes in SW480 cells. (a), Real-time RT-PCR analyses of gene expression with the Signal Transduction Pathway Array from SA Biosciences. The genes with twofold changes are plotted. Upregulated genes are shown in red, and downregulated genes in green. (b), Real-time RT-PCR analyses for the genes identified in the miniarray. β-actin was used as an internal loading control. **P<0.01.

To gain insight into the molecular mechanism by which DHX32 contributed to tumorigenicity of CRC, a SA Biosciences 96-well Signal Transduction Pathway Finder PCR Array plate with pre-coated primer pairs was used to detect gene expression changes of 10 commonly studied signal transduction pathways. The microarray analysis was carried out with DHX32-depleted cells and control cells. Scatter plot depicting expression profiles of all the 84 tested genes in DHX32-depleted versus control cells was displayed in Figure 5a. The genes that exhibited at least twofold increases or reduces were indicated by colored circles. The expression profiles validated by real-time RT-PCR were shown in Figure 5b. Notably, DHX32 depletion attenuated expression of 5 genes. Among them, *WISP1* (*WNT-inducible signaling pathway protein 1*), *MMP7* (*matrix metallopeptidase 7*), and *VEGFA* (*vascular endothelial growth factor A*) are within the Wnt pathway. Additionally, the anti-apoptotic genes *Bcl-2* (*B cell leukemia/lymphoma 2*) and *CA9* (*carbonic anhydrase IX*) were downregulated 4 and 10 fold respectively in DHX32-depleted cells compared to control cells. In contrast, *ACSL5* gene expression was increased in DHX32 depleted cells, which was implicated in the activation of PPAR. Therefore, the gene expression profile changes suggest a positive roles of DHX32 in cell proliferation, migration, and invasion possibly via activation of the Wnt signaling pathway and a negative role in apoptosis.

## Discussion

With a few exceptions, the biochemical activities and biological roles of RNA helicases, including DHX32, are not very well characterized. Our previous work shows that *DHX32* is up-regulated in CRC compared to its adjacent normal tissues and the level of *DHX32* expression is associated with the progression of CRC^14^. In this report, we further demonstrated that expression of DHX32 was elevated in human CRC cells at both mRNA and protein levels. We also showed that upregulation of DHX32 promoted CRC cell proliferation, migration, and invasion, as well as inhibited apoptosis. The data indicate that upregulation of DHX32 favors CRC progression and metastasis.

There are examples of dysregulation of human RNA helicase expression in various types of cancer^16^. Furthermore, it has been suggested that RNA helicase can either regulate the activity with or without direct interactions with the molecules that are implicated in cancer. Among them, DDX5, DHX9 and DDX17 have been shown to be involved in transcription regulation^17^,^18^,^19^. To date, however, although several reports have shown that DHX32 contributes to cancer development, the molecular mechanism underlying the oncogenic activities of DHX32 are still elusive.

In this study, we utilized the RT^2^ *profiler*^TM^ PCR Array to determine the specific signaling pathways and the gene expression profiles that were regulated by DHX32. It was found that multiple genes overexpressed in CRC cells were downregulated by depletion of DHX32 expression. Among the 10 signaling pathways analyzed, the most notable changes were in the Wnt signaling pathway. Wnt signaling is a well-characterized pathway that promotes progression and metastasis in many types of cancers. Aberrant activation of the Wnt pathway, either through the genetic or epigenetic alterations, has been found to contribute to tumorigenesis. In fact, it has been reported that aberrant Wnt signaling is an early progression event in 90% of CRC^20^,^21^,^22^,^23^. As described in the Results section, we observed that depletion of DHX32 down-regulated *WISP1*, *MMP7* and *VEGFA* expression at the mRNA level, implying that DHX32 contributed to CRC progression through upregulating the Wnt pathway. Our results also suggested that DHX32 promoted migration and invasion activities of CRCs. The results are in accordance with our previous report that the upregulated expression of *DHX32* in colorectal cancer tissues is associated with lymph gland metastasis^14^.

In addition, our data showed that depletion of DHX32 in CRC cells increased expression of pro-apoptotic molecule *ACSL-5* and reduced anti-apoptotic molecules *Bcl-2* and *CA9*. Several studies show that ACSL-5 is expressed in mitochondria and its activities are associated with increased susceptibility of apoptosis and cell death pathways^24^,^25^. Recent studies also reveal that Bcl-2 is related to mitochondria-apoptotic regulation and modulation^26^,^27^. CA9 is overexpressed in a wide variety of human cancers. Inhibition of CA9 decreases cell proliferation and induces apoptosis^28^,^29^. DHX32 is the first putative RNA helicase in the DHX family that is localized in the mitochondria^13^. The mitochondria residence of DHX32 suggests a potential role of DHX32 in mitochondria-mediated apoptosis through processing mitochondrial RNA.

5-FU is widely used in treating CRC. However, cancer cells frequently evade drug-induced death signals. As a result, chemoresistance is the major impediment of 5-FU-based therapies. Therefore, to identify new molecular targets for 5-FU treatments will provide new approaches to increase 5-FU efficacy for CRC treatment. Recent studies showed that the Wnt pathway plays an important role in chemotherapy resistance of various malignancies^30^,^31^. Our data showed that overexpression of DHX32 in CRCs protected the cells from cell death induced by 5-FU. Interestingly, we also noticed that 5-FU treatment decreased DHX32 expression at the mRNA and protein levels in a dose-dependent manner (Fig. S3). Thus, concurrently treating the patients with 5-FU and DHX32 inhibitors can be a viable option for CRC chemotherapies.

In conclusion, this is the first report demonstrating that DHX32 promotes cell proliferation, migration, and invasion, as well as reduces sensitivity to chemotherapy reagents in CRC, via modulating expressions of gene involved in cancer cell growth, migration, and survival. It is worthy to determine whether DHX32 can be used as a novel target for CRC treatment in conjunction with other chemotherapy reagents.

## Methods

### Cell culture and reagent

Human colorectal cell CCD-18Co and human colorectal carcinoma LS174T cell were grown in high-glucose Dulbecco's modified Eagle's medium (DMEM, Gibco), and human colorectal cell NCM460 and human CRC SW480, SW620, and HCT-8 cells were grown in the RPMI-1640 medium (Gibco), with supplements of 10% (v/v) fetal bovine serum (Gibco) and 100 units/ml streptomycin and penicillin (Millipore), at 37 °C.

The chemical reagents 5-FU was purchased from Sigma; puromycin and blasticidin from InvivoGen. The anti-DHX32 antibody was purchased from Sigma; anti-β-actin antibody from Santa Cruz.

### DNA constructs

DHX32 expression plasmid was constructed by sub-cloning a PCR product of human DHX32 cDNA into pLOC vector and the sequence of the cDNA was confirmed.

For gene silencing, five independent short hairpin RNA (shRNA) targeted to DHX32 were cloned into a pGIPZ vector. The sequences of DHX32 shRNAs are 5′-CGCCATATTCATCACGTTATTA-3′ (F-1), 5′-CGGTTGTTCCTTTGTATCCAAA-3′ (B-11), 5′–AAAGGAAAACACTCTAAACATA-3′ (F-11), 5′-AATCCACAACTCTCGAAGTCTA-3′ (G-8), and 5′- ACCCTGTGTCAACAATGAATAA-3′ (E-10).

### Stable cell line generation

For establishment of stable DHX32-depleted cell line, SW480 cells were cultured for 24 hours to 60%–80% confluence before transfected with the DHX32-shRNA constructs using the FuGENE HD Transfection Reagent (Promega). The pGIPZ empty vector was used as a negative control. Twenty four hours post-transfection, the cells were selected with 2 μg/ml puromycin for two weeks. The efficiency of the depletion was determined by real-time RT-PCR and western blot analysis. The most efficient shRNA (G-8) was chosen to deplete DHX32 thorough out the study.

pLOC-DHX32 clone and pLOC empty vector was stably transfected using FuGENE HD Transfection Reagent and selected with 20 μg/ml blasticidin for two weeks. Blasticidin resistant single clones were isolated and two of these independent clones were successfully established, which were designated as SW480-DHX32 and SW480-NC, respectively. The DHX32 expression in these cell lines was determined by real-time RT-PCR and western blot.

### Quantitative real-time RT-PCR

Total RNA was extracted from cells using Trizol (TIANGEN) according to the manufacturer's protocol and the cDNA was synthesized using the RevertAid First Strand cDNA Synthesis Kits (Fermentas). Quantitative real-time RT-PCR was then performed using gene-specific primers as described in our previous study^14^. The expression of target transcripts was normalized to the *β-actin* internal control, and relative changes of gene expression were determined using the 2^−ΔΔCt^ method. The primers for *DHX32* are 5′-GTCTTTCCATCCACTACCAGCAC-3′ (forward) and 5′-ATGATGACCCCATAGCTACCCAA-3′ (reverse); for *β-actin* are 5′-TCACCCACACTGTGCCCATCTACGA-3′ (forward) and 5′-CAGCGGAACCGCTCATTGCCAATGG-3′ (reverse); for *ACSL5* are 5′-TGGCTATCTTACAAACAGGTGTC-3′ (forward) and 5′-TCCACTCTGGCCTATTCTGAG-3′ (reverse); for *Bcl2* are 5′-GGTGGGGTCATGTGTGTGG-3′ (forward) and 5′-CGGTTCAGGTACTCAGTCATCC-3′ (reverse); for *CA9* are 5′-GGATCTACCTACTGTTGAGGCT-3′ (forward) and 5′-CATAGCGCCAATGACTCTGGT-3′ (reverse); for *MMP7* are 5′- ATGTGGAGTGCCAGATGTTGC-3′ (forward) and 5′- AGCAGTTCCCCATACAACTTTC-3′ (reverse); for *VEGFA* are 5′-AGGGCAGAATCATCACGAAGT-3′ (forward) and 5′-AGGGTCTCGATTGGATGGCA-3′ (reverse); for *WISP1* are 5′-AGGAACTGCATAGCCTACACA-3′ (forward) and 5′- TGGTACACAGCCAGACACTTC-3′ (reverse).

### Immunoblotting

Immunoblotting assay was carried out as described previously^32^. Briefly, cells were lysed using ice-cold lysis buffer TNTE 0.5% (0.5% Triton X-100, 50 mM Tris-HCl, pH 7.5, 150 mM NaCl, 1 mM EDTA) containing 10 μg/ml pepstatin A, 10 μg/ml leupeptin, and 1 mM PMSF. The total cell lysates were then applied to immunoblotting assays using appropriate antibodies and detected by Chemiluminescent HRP substrates (Millipore). The gels in the figures have been run under the same experimental conditions.

### Cell proliferation assays

The Cell Counting Kit-8 (CCK-8) (Dojindo) was used to measure cells proliferation. 3 × 10^3^ SW480 cells per well were seeded in 96-well plates and incubated for 24, 48, 72, and 96 hours, respectively. The cells were then incubated with the CCK-8 reagent (10 μl per well) for 2 hours prior to measure the absorbance of 450 nm with an ELISA plate reader (Thermo).

### Colony formation assays

Cells were seeded in 6-well plates at a density of 500 cells per well. After incubating for 12 days, the colonies were fixed with methanol and stained with Giemsa. Only those colonies containing more than 50 cells were counted.

### Wound healing assay

Cells were seeded in 6-well plates at a density of 10^5^ cells per well and incubated for 24 hours. Then a 20 μl pipette tip was used to scratch a linear wound in the cell monolayer. Photos were taken at 0, 24, 48, and 72 hours after the scrapping, respectively.

### Cell migration assay

Chemotactic migration of SW480 cells was examined using the Transwell Chember Assay. Cells were serum-starved for 24 hours and then plated at the density of 10^5^ cells per well in the serum-free medium. The RPMI 1640 medium containing 10% FBS was added into the bottom chamber as a chemoattractant. After incubation for 24 hours, non-migrating cells were removed from the upper chamber with a cotton swab and cells migrated through the membrane were fixed with 4% formaldehyde and stained with crystal violet staining solutions. Cell numbers in five randomly selected fields were counted under a microscope (Leica) with 200 × magnification.

### Matrigel invasion assay

The Matrigel Invasion chambers with 8 micron Matrigel-coated filters (BD BioCoat) were used to study the cell invasion activity. Briefly, cells were serum starved for 24 hours and then seeded in the upper compartment of Matrigel-coated inserts at a concentration of 5 × 10^5^ cells per well. The medium containing 10% FBS was added into the bottom chamber. After incubation for 24 hours, non-invaded cells in the upper chamber were removed. Cells that invaded through the membrane to the lower surface were counted under a microscope (Leica) with 200 × magnification and photographed.

### Apoptosis analysis

Cells cultured in 6-well plates were treated with the indicated chemicals, trypsinized, and collected in 1.5 ml tubes. After centrifugation, the cells were resuspended in 1 × binding buffer. The cell suspensions were then used for apoptosis analyses with the PE Annexin V Apoptosis Detection Kit (BD Biosciences) according to the manufacturer's instruction. After incubation at room temperature in the dark for 15 minutes, an excess of 1 × binding buffer was added to a final volume of 500 μl. A total of 10,000 cells were examined by flow cytometry using a BD Influx cell sorter (BD Biosciences). Cells were gated on side scatter and forward scatter to exclude debris. Annexin V/7-AAD double positive and Annexin V–positive/7-AAD–negative cells were defined as apoptotic cells.

### Gene array analysis

The Human Signal Transduction PathwayFinder^TM^ RT^2^ *Profiler*^TM^ PCR Array (SA Biosciences) was used, which includes cDNAs for 84 key representative genes of 10 signal pathways. Briefly, total RNAs were extracted from control and DHX32 depleted cells using the RNeasy Mini kit (Qiagen) according to the manufacturer's protocol. RNA integrity and quality was determined prior to gene expression analyses. For the cDNA synthesis, 5 μg of total RNA was reverse transcribed (RT) to first-strand cDNA according to the protocol provided by the RT^2^ Easy First Strand Kit (SA Biosciences). The resultant cDNA was diluted and mixed with the SA Biosciences RT^2^ qPCR Master Mix and water to a total volume of 2.7 ml. The reaction mixture (25 μl per well) was loaded into a 96 well plate for real-time PCR analyses performed on an ABI7500 (Life Technologies) PCR machine following the instructions provided by the PCR array kit. The mRNA levels were normalized against housekeeping genes included in the PCR array (β-glucuronidase; hypoxanthine guanine phosphoribosyl transferase 1; heat shock protein 90 kDa α; glyceraldehyde-3-phosphate dehydrogenase; and β-actin). Gene expression levels were calculated using the RT^2^ Profiler PCR Array Data Analysis Template v3.3 software and were expressed as folds of control cells. Unsupervised clustergram/heat map were generated by using the RT^2^ PCR array data analysis web portal (http://www.sabiosciences.com/pcrarraydataanalysis.php).

### Statistical analysis

Data are expressed as mean ± SD of triplicate samples. The data significance was evaluated by using Student's t-test. P<0.05 was considered a statistically significant difference.

## Author Contributions

Z.Z. conceived experiments. F.W. and H.L. wrote the main manuscript text. H.L. and W.L. prepared figures. Z.F., X.L., J.L., Y.B., L.L. and H.Y. performed experiments. Y.P. helped J.L. in biochemical experiments.

## Supplementary Material

Supplementary Information Supplementary information
