# Supplementary material for: Overexpression of DHX32 contributes to the growth and metastasis of colorectal cancer
Source: Sci Rep. 2015 Mar 18;5:9247. doi: 10.1038/srep09247 (PMC4363870; doi:10.1038/srep09247)

Supplementary Information

Overexpression of DHX32 contributes to the growth and metastasis of colorectal cancer

Huayue Lin[1,#], Wenjuan Liu[1,#], Zanxi Fang[1], Xianming Liang[1], Juan Li[1], Yongying Bai[1], Lingqing Lin[1], Hanyu You[1], Yihua Pei[3], Fen Wang[4,*], and Zhong-Ying Zhang[1,2,*]

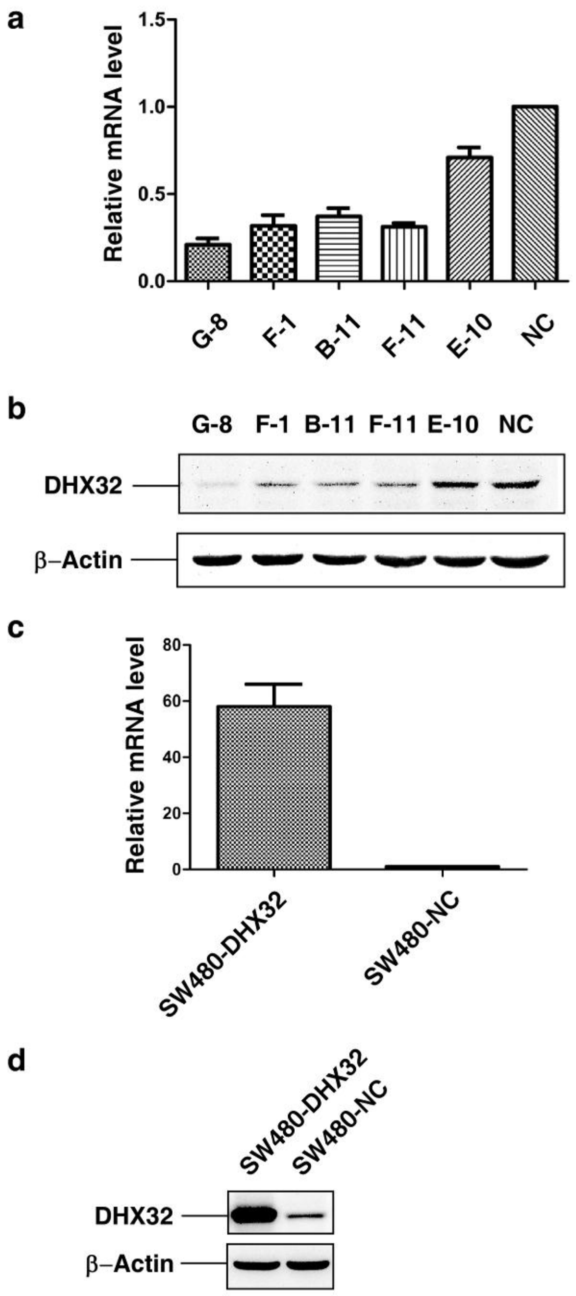

Figure S1. Stable depletion or overexpression of DHX32. Real-time RT-PCR and western blot analyses showed that expression of DHX32 was reduced (a, b) or increased (c, d) by shRNA or cDNA transfection, respectively. β-actin was used as a loading control. NC, vector only as a negative control.

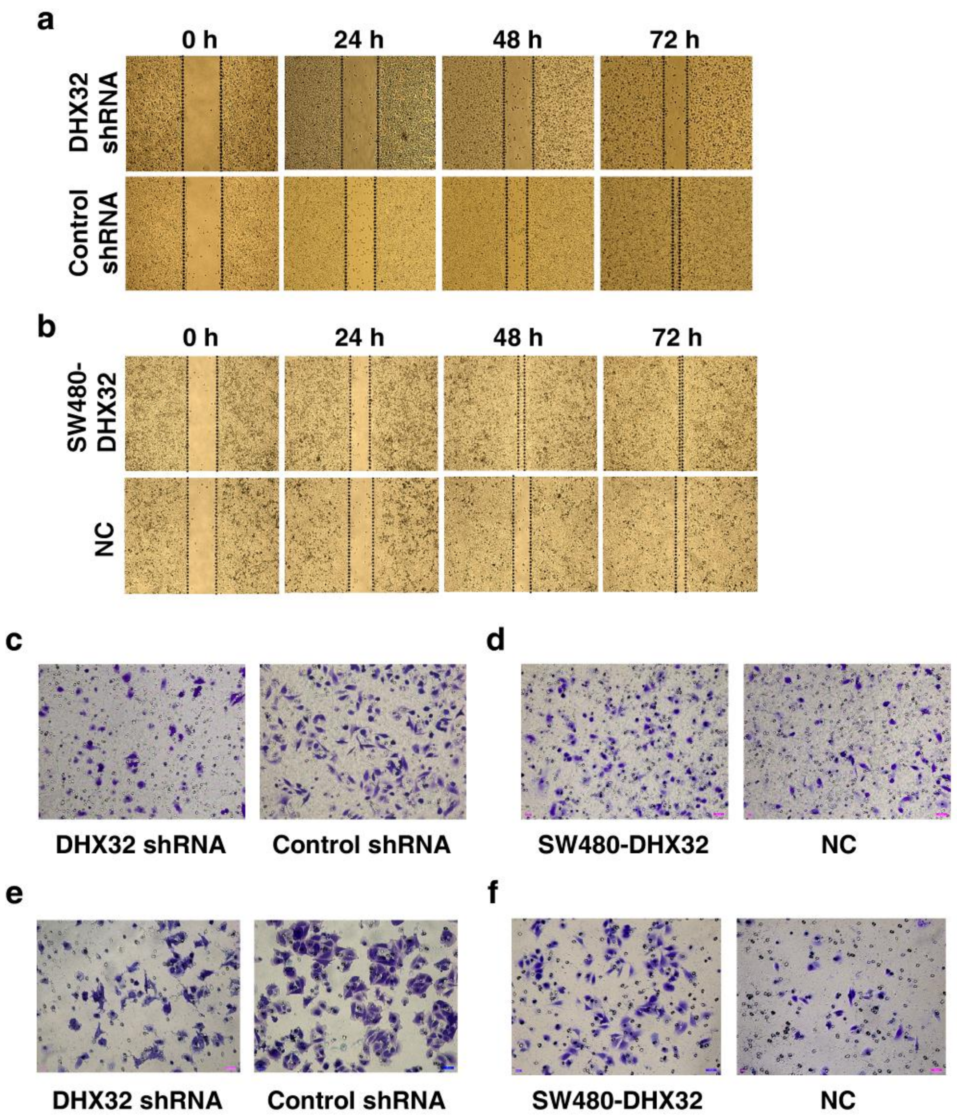

Figure S2. DHX32 promoted colorectal cancer cell migration and invasion. **a&b**, The cell motility was analyzed by the scratch assays as described in Methods. Cell migration was performed using the Transwell chambers(c, d) and invasion through the Matrigel-coated filters(e, f). After 24 hours of incubation, DHX32-depleted cells and the control cells remained in the upper chambers were removed and cells migrated to the lower chambers were fixed, stained, and counted in six random fields with 200× magnification. For DHX32-overexpressed cells and the control cells, the harvest time was 18 hours. Representative images were shown.

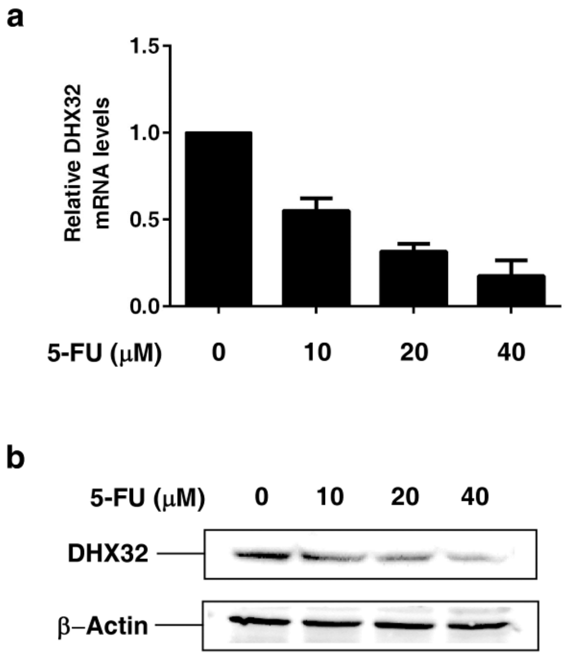

Figure S3. 5-FU decreased DHX32 mRNA and protein levels. **a**, 5-FU inhibited DHX32 expression at mRNA level. SW480 cells were treated with different doses of 5-FU (10, 20, 40 μM) for 12 hours, and DHX32 mRNA levels were determined using RT-qPCR. **b**, 5-FU suppressed DHX32 expression at protein level. SW480 cells were treated with different doses of 5-FU (10, 20, 40 μM) for 12 hours, and the protein levels of DHX32 were detected by immunoblotting. β-actin levels were used as a loading control.

The full-length blots in Fig.1 b

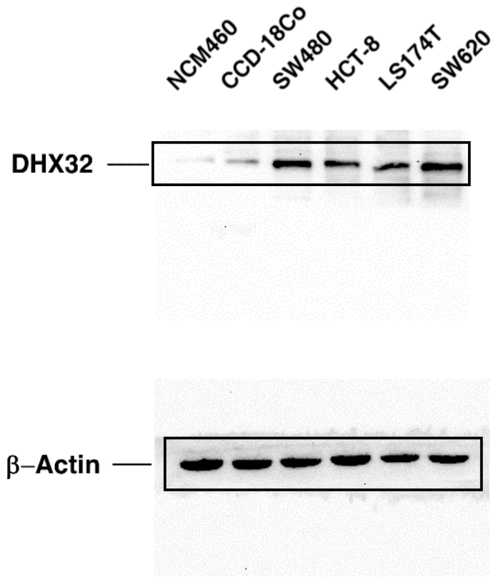

The full-length blots in Fig.3 c

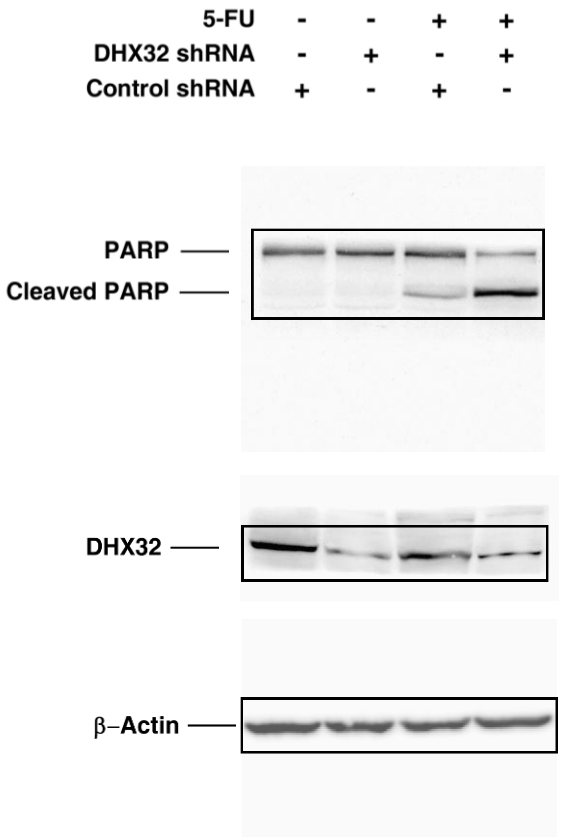

The full-length blots in Fig.3 d

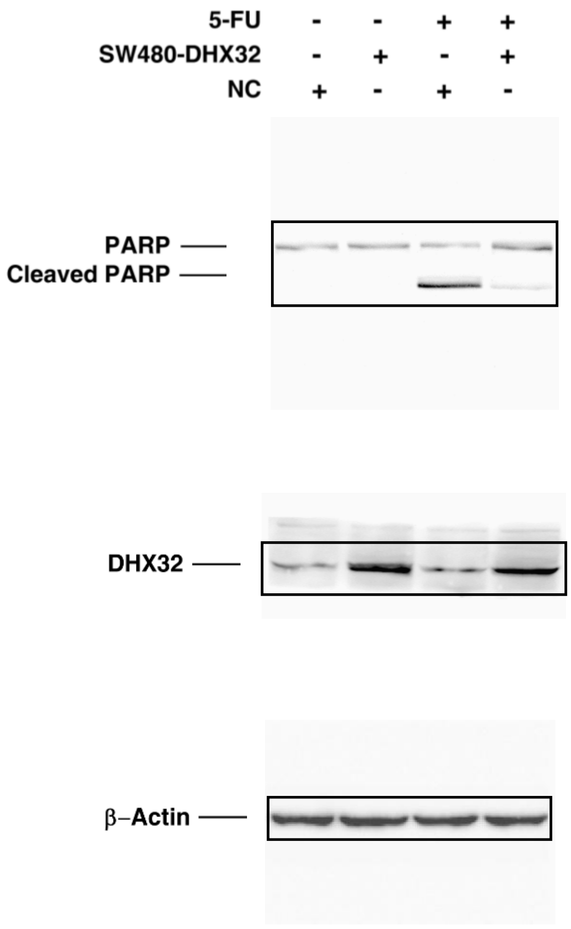

Supplement: Supplementary Information [file srep09247-s1.pdf]
